# Adipose-Derived Mesenchymal Stem Cells for Treatment of Airway Injuries in A Patient after Long-Term Exposure to Sulfur Mustard 

**DOI:** 10.22074/cellj.2016.4874

**Published:** 2016-12-21

**Authors:** Amir Nejad-Moghaddam, Soheila Ajdari, Eisa Tahmasbpour, Hassan Goodarzi, Yunes Panahi, Mostafa Ghanei

**Affiliations:** 1 Chemical Injuries Research Center, Baqiyatallah University of Medical Sciences, Tehran, Iran; 2 Department of Immunology, Pasteur Institute of Iran, Tehran, Iran

**Keywords:** Mesenchymal Stem Cells, Transplantation, Sulfur Mustard, Airway Remodeling

## Abstract

**Objective:**

Sulfur mustard (SM) is a potent mutagenic agent that targets several organs, particularly lung tissue. Changes in morphological structure of the airway system are associated with chronic obstructive pulmonary deficiency following exposure to SM. Although numerous studies have demonstrated pathological effects of SM on respiratory organs, unfortunately there is no effective treatment to inhibit further respiratory injuries or induce repair in these patients. Due to the extensive progress and achievements in stem cell therapy, we have aimed to evaluate safety and potential efficacy of systemic mesenchymal stem cell (MSC) administration on a SM-exposed patient with chronic lung injuries.

**Materials and Methods:**

In this clinical trial study, our patient received 100×106cells every 20 days for 4 injections over a 2-month period. After each injection we evaluated the safety, pulmonary function tests (PFT), chronic obstructive pulmonary disease (COPD) Assessment Test (CAT), St. George’s Respiratory Questionnaire (SGRQ), Borg Scale Dyspnea Assessment (BSDA), and 6 Minute Walk Test (6MWT). One-way ANOVA test was used in this study which was not significant (P>0.05).

**Results:**

There were no infusion toxicities or serious adverse events caused by MSC administration. Although there was no significant difference in PFTs, we found a significant improvement for 6MWT, as well as BSDA, SGRQ, and CAT scores after each injection.

**Conclusion:**

Systemic MSC administration appears to be safe in SM-exposed patients with moderate to severe injuries and provides a basis for subsequent cell therapy investigations in other patients with this disorder (Registration Number: IRCT2015110524890N1).

## Introduction

Sulfur mustard (IUPAC ID: bis(2-chloroethyl) sulfide, SM) is a potent vesicating blistering chemical warfare agent first used in World War I and subsequently by Iraq against Iran in the 1980s (1). The molecular mechanisms of its action, chemistry, and genotoxicity, as well as the pathogenesis and histopathology of SM injuries are widely described (2). SM primarily affects the skin, eyes, and lungs (3). During the Iran-Iraq War, about 100,000 people were exposed to this chemical warfare agent. Unfortunately more than 40000 patients still suffer from the chronic effects of SM (4, 5). Changes in morphological structure of the airways following exposure to SM in patients with chronic obstructive pulmonary disease (COPD) can occur (6). Radiological and pathological findings confirm remodeling of the airway system such as thickening of the bronchial wall and narrowing of the lumen. It has been suggested that clinical symptoms and structural changes in bronchial walls of SM injuries are relatively similar to some characteristics of asthma patients (7). Current regimes for respiratory disorder consist of bronchodilators, N-acetyl cysteine, and antibiotics (8). In addition to oxygen therapy (9), antibiotics, suppressant agents, prednisolone (10), membrane stabilizers, antioxidants (11), macrolides, and interferon (12) are other drugs used to treat chemical injuries. These current therapies are not suitable for patients with SM because the airway structure is destroyed and requires a regeneration method for airway treatment. 

The past decade has witnessed promising results for cell therapy with somatic stem cells in different models of lung injuries such as acute respiratory distress syndrome (13). A collection of data from several clinical trials has also shown that transplantation of adipose derived mesenchymal stem cells (ADMSCs) to patients is safe and non- toxic (14). MSCs have exerted a beneficial effect in both phase 1 and 2 clinical trials of graft-versus- host disease (GVHD) (15). A clinical trial of MSCs in COPD showed non-significant improvement in pulmonary function or frequency of exacerbations. Weiss et al. (16) injected allogeneic bone marrow derived MSCs (BMSCs) into COPD patients. Interestingly, their study illustrated that intravenous injection of MSCs was safe in these patients. Additionally, another study on patients with idiopathic pulmonary fibrosis (IPF), injected autologous MSCs derived from lipoaspirations into the endobronchial area. Results showed that endobronchial administration of ADMSCs was safe for these patients (17).

In addition to adipose and BMSC, there is an increasing interest in lung-resident MSCs. Recent studies have focused on lung resident MSCs isolated from bronchoalveolar lavage (BAL) fluid of lung-transplanted patients (18). According to recent data, MSCs can be isolated from both central and peripherally located lung tissue of lung-transplanted patients. Isolated MSCs can form bone, cartilage and muscle, as well as fat (19). These cells have been variously known as pre-adipocytes, stromal cells, and adipose-derived adult stem cells (20). A large number of adipose stromal cells (ASCs) can be derived from lipoaspirate, as the waste product of liposuction surgery. For example, processing of 300 ml lipoaspirate routinely yields 1×10^7^ADMSCs with >90% cell viability (21). Compared with BMSCs, ADMSCs are easier to culture and grow more rapidly (22). They can be cultured for a long time before senescence compared to BMSCs (23). There is no effective strategy for treatment of pulmonary disorders in SM-exposed patients. Possibly, injection of MSCs may assist with improvement of respiratory problems in these patients. Therefore, we have considered, for the first time, the safety and potential efficacy of ADMSCs administration on an SM-exposed patient with a chronic lung injury.

## Materials and Methods

We used the following materials in this clinical trial study: collagenase A type I (Sigma, USA), fetal bovine serum (FBS, Gibco, USA), MEM Alpha 1x (Gibco, USA), L-glutamine (Gibco, USA), antibiotic-antimycotic solution (Gibco, USA), trypsin-EDTA (Gibco, USA), CD90-fluorescein isothiocyanate (FITC), CD73-phycoerythrin (PE), CD105-PE, CD34-FITC, CD45-PE, CD11b-FITC, CD44-FITC (Sigma-Aldrich, USA), colcemid solution (Invitrogen, USA), and dimethylsulfoxide (DMSO, Gibco, USA). One-way ANOVA test was used in this study which was not significant (P>0.05).

### Patient

We conducted this clinical trial at the Chemical Injuries Research Center, Baqiyatallah University of Medical Sciences, Tehran, Iran (Ethical code: IR.BMSU.REC.1393.32). In this study, we considered the therapeutic effect of MSCs in an SM-exposed male patient. Our patient had a documented encounter with SM during the Iran-Iraq war. The patient signed an informed consent before study. He was selected according to the following criteria: i. The patient had a forced expiratory volume in 1 second (FEV1) of moderate (>50 to <65) to severe (>40 to <50), ii. Absence of contraindications for spirometry (hemoptysis, cerebral arterial aneurysm or aortic aneurysm, pulmonary embolism, uncontrolled blood pressure, recent pneumothorax, history or any recent thoracic event, recent stroke, and iii. No problems with coagulation. Exclusion criteria were as follows: i. Simultaneous participation in another study, ii. Smoker, iii. The existence of pneumonia during the study, iv. Transfusion reaction, and v. Other underlying conditions such as cardiovascular disease, hypertension, or diabetes.

### Isolation and culture of adipose derived mesenchymal stem cells 

A liposuction aspirate protocol was used to obtain 200 mL of abdominal adipose tissue under local anesthesia. The lipoaspirate was washed with phosphate-buffered saline (PBS) to remove tissue debris after which 100 mL of PBS that contained 0.1% w/v collagenase A type I (Sigma, USA) was added to the isolated tissue, followed by incubation at 37˚C for 60 minutes. Collagenase activity was neutralized using MEM medium (Gibco, USA) along with 10% FBS (Gibco, USA). Cell pellets were resuspended in culture medium after centrifugation at 2000 rpm for 10 minutes and then transferred to culture flasks for 72 hours at 37˚C and 5% CO_2_. The culture medium in the flasks was changed every 3 days and cells were passaged 3 times. Lipoaspiration procedure and cell preparation were carried out in Department of Regenerative Biomedicine, Royan Institute.

### Flow cytometry analysis

In order to analyze the cell surface antigen expression, we used trypsin-EDTA to harvest 5×105 fresh passage-3 cells. Cells were centrifuged at 100 g for 1 minute, resuspended in stain buffer (PBS, 2% FBS) and incubated on ice for 10 minutes. Trypsin was neutralized by centrifugation; isolated cells were washed twice with PBS and resuspended in stain buffer. Cells were incubated in the dark for 30 minutes. After incubation, the cells were labeled with the following anti-human monoclonal antibodies (MAbs) conjugated to fluorochromes: anti-CD90-FITC, CD73-PE, CD11b-FITC, CD34- FITC, CD44-FITC, CD45-PE, and CD105-PE (Sigma-Aldrich, USA). The frequencies of all immunolabeled cells were analyzed by a FACS Canto II flow cytometer (BD Biosciences, USA), in which approximately 500,000 events were assessed. Data were analyzed by FlowJo software (version 10.0).

### Karyotype analysis for detection of abnormalities

We used standard giemsa staining procedure and chromosome preparations were obtained from 80% confluent cells. The cells were treated with Colcemid solution (Invitrogen) in order to stop microtubule formation. The mitotic arrested cells were then harvested using trypsin-EDTA. The cells were extracted and then immersed in 75 mmol/l KCl for 30 minutes at room temperature. Finally, the cells were centrifuged at 400×g for 10 minutes. The supernatant was replaced with fixative solution and the suspension was spread over slides for microscopic examination and imaging. At least 15 metaphase spreads were analyzed. The karyotypes were observed by light microscope (100X, Nikon) using CytoVision software (version 7.2). 

### Freezing and storage of adipose-derived mesenchymal stem cells 

We harvested ADMSCs once they reached 90% confluency. In order to collect the cells, the culture medium was removed and replaced by sterile PBS. After three minutes, PBS was replaced by trypsin-EDTA solution. The cells and this solution were incubated at 37˚Cfor 5 minutes. Complete medium (MEM with 10% FBS) was added to inactivate the trypsin, and the solution was centrifuged at 1500 rpm for 5 minutes. The cell pellet was resuspended in cryopreservation medium (80% FBS, 10% DMSO, and 10% MEM medium) with a final concentration of 5×10^6^cells/ml and aliquoted into cryovials. The vials were frozen overnight at -80˚C and then transferred into a liquid nitrogen container for long-term storage until they were thawed for the injections.

### Injection of mesenchymal stem cells and study protocol

**Fig.1:**
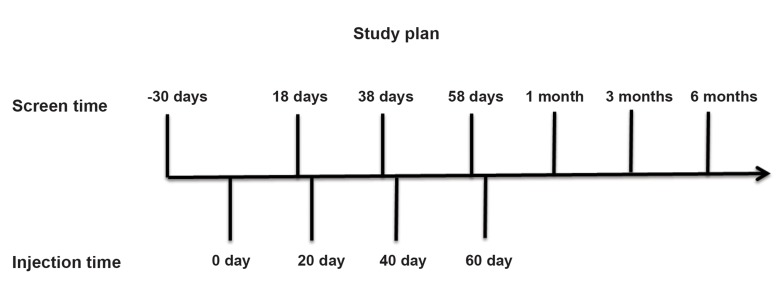
Schematic study design. Mesenchymal stem cells (MSCs) were injected 4 times each 20 days. The patient was evaluated 20 days before the first infusion and then 2 days before the second, third, and fourth injections. He was also evaluated at days 90, 150, and 240 after the first infusion.

Our patient received 100×10^6^cells every 20 days for a total of 4 injections within a 2-month period. He was screened 7 times for evaluation of physical activities and respiratory quality (Fig.1). MSCs were injected intravenously along with 300 ml normal saline at a maximum rate of 2×106 cells/minutes. Each infusion took approximately 30 minutes until completion. After each injection, our patient remained at the hospital for at least 6 hours as the recovery time. We evaluated the efficacy of the injections in the patient according to the following parameters: pulmonary function tests (PFTs) [FEV1, forced vital capacity (FVC), FEV1/FVC], total lung capacity (TLC) by body plethysmography, single-breath carbon monoxide diffusing capacity (CO diffusion), exercise performance [6 minute walk test (6MWT)] (24), Borg Scale Dyspnea Assessment (BSDA) (25), COPD Assessment Test (CAT), St. George’s Respiratory Questionnaire (SGRQ) (26), and a comprehensive safety evaluation.

## Results

**Fig.2:**
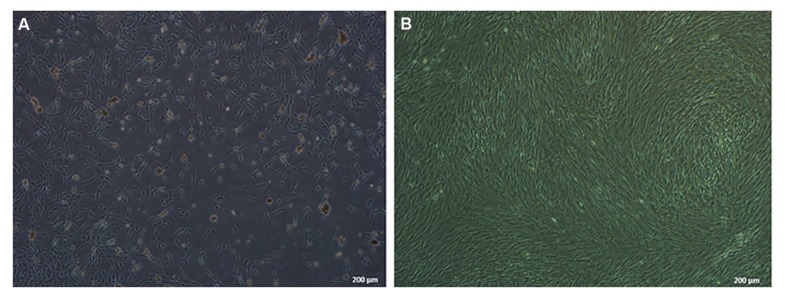
Mesenchymal stem cells (MSCs) were isolated and cultured until 90% confluency for the injections. A. Stem cells shown at day 4 after beginning to start culture with 25% confluency and B. Cells reached more than 90% confluency by the third passage.

Cell confluency in first passage was 25% (Fig.2A), which increased in subsequent, continuous passages. We minimized the number of passages in order to decrease the chances of chromosomal mutations. We isolated ADMSCs that had >90% confluency for injection purposes (Fig.2B).

**Fig.3:**
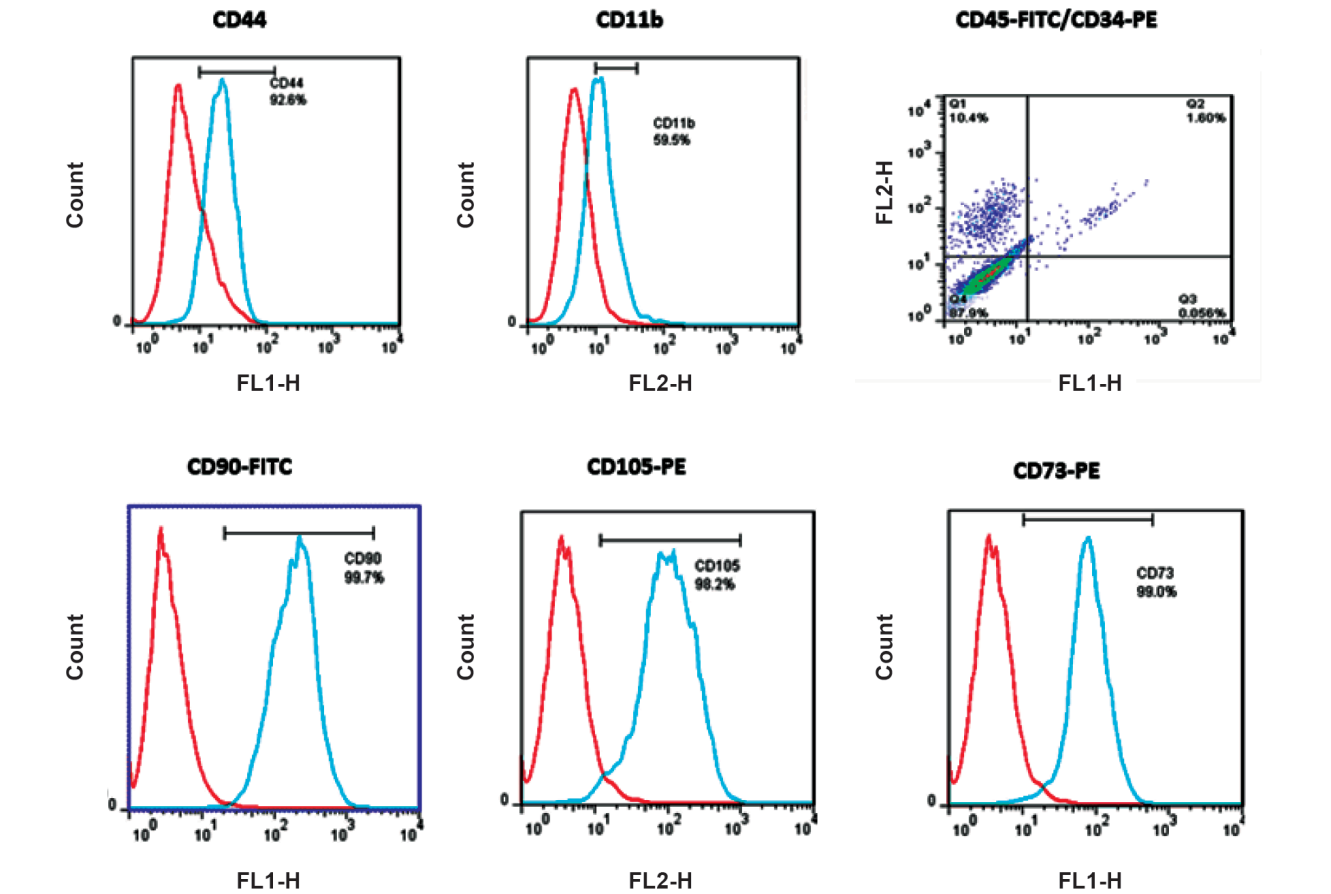
Phenotype of mesenchymal stem cells (MSCs) as determined through cell surface markers [(CD90, CD73, CD44 and CD105)+, (CD34, CD45)−, and CD11b−] by flow cytometric analysis. Data were analyzed with FlowJo software. The existence of CD73, CD90, CD105, and CD44 markers demonstrated that the cultured cells were adipose derived MSCs (ADMSCs).

 CD markers (CD73, CD90, CD105, and CD44) demonstrated that the cultured cells were indeed ADMSCs (Fig.3). These CD markers are specific for ADMSCs, as identified by specific MAbs.

**Fig.4:**
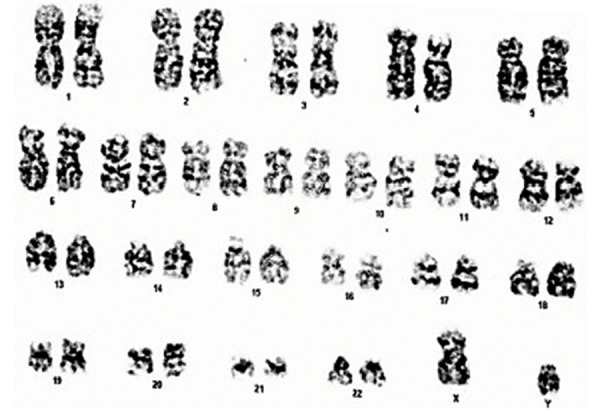
Karyotyping of human adipose derived mesenchymal stem cells (ADMSCs). Karyotype analysis of passage-3 ADMSCs before cell freezing. Karyotyping showed that the MSCs were normal and could be used for the injections.

The karyotyping method was applied to consider any possible abnormalities in cells before the ADMSCs injections. Our data showed normal MSCs (Fig.4). In case of chromosomal abnormalities, we would have cancelled injections and repeated the sampling process.

### Efficacy outcomes

**Fig.5:**
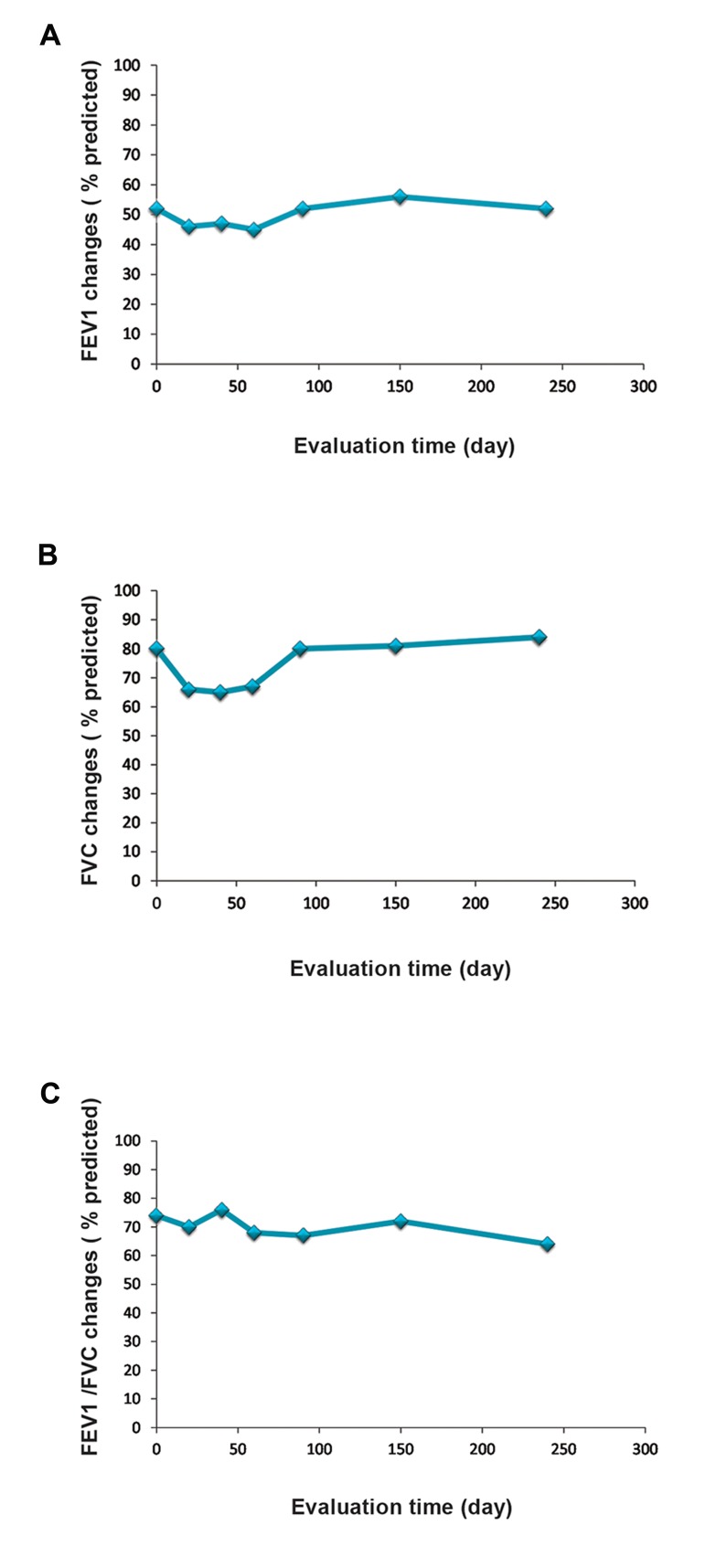
Comparison of lung function tests before and after mesen- chymal stem cell (MSC) injections. A. Forced expiratory volume in 1 second (FEV1) score, B. Forced vital capacity (FVC) score, and C. FEV1/FVC. Although pulmonary function tests (PFTs) showed no significant improvement, we observed a trend toward im- provement after the second injection.

There were no statistically significant differences observed in PFTs (FEV1, FVC, and FEV1/FVC %) during 9 months (Fig.5A-C). However, we found a trend toward improved PFTs after the second injection. Our data indicated a reduced volume for diffusing capacity or transfer factor (TLco) of the lung for carbon monoxide. There was no significant difference in TLC, residual volume (RV) and maximum expiratory flow (MEF) 25-75 after the injections (Table 1).

**Fig.6:**
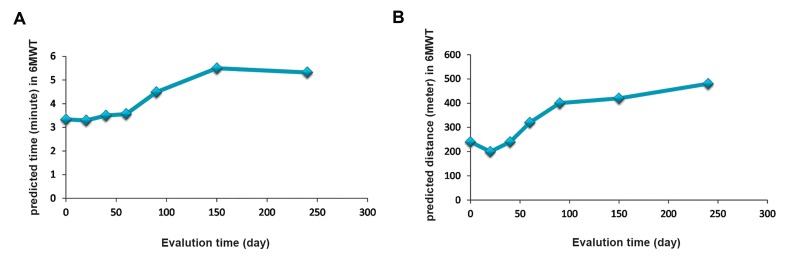
Comparisons of the 6 minute walk test (6MWT) on A. Base predicted time and B. Predicted distance during mesenchymal stem cell (MSC) therapy. The 6MWT showed improvement after each evaluation time.

**Fig.7:**
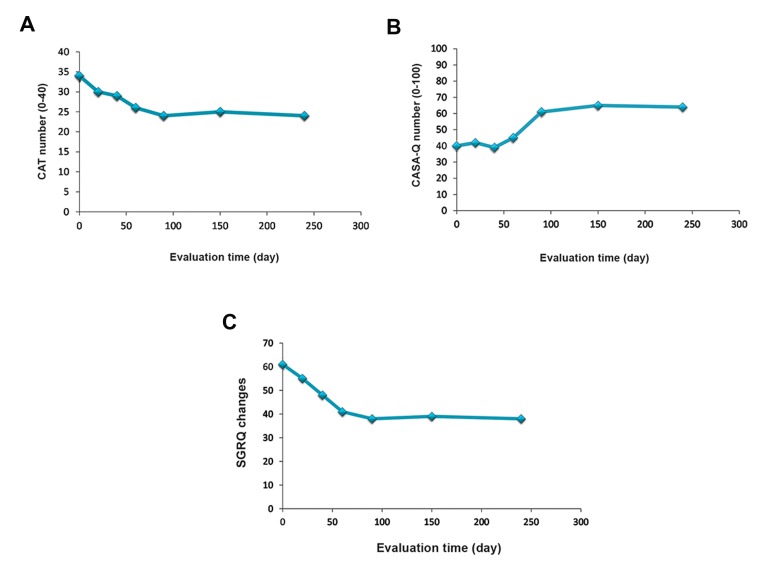
Evaluation of chronic obstructive pulmonary disease (COPD) A. Assessment test (CAT), B. Cough and Sputum Assessment Ques- tionnaire (CASA-Q), and C. St. George’s Respiratory Questionnaire (SGRQ) scores before and after cell therapy. This data demonstrated improved respiratory quality after each assessment.

Interestingly, we observed a significant improvement in the 6MWT (predicted time and distance) after treatment with MSCs from day 0 to month 9 (Fig.6A, B). There were no significant differences in oxygen saturations in the 6MWT during the study visits. CAT (Fig.7A), Cough and Sputum Assessment Questionnaire (CASA-Q) (Fig.7B), and SGRQ scores significantly improved after the injections (Fig.7C).

**Fig.8:**
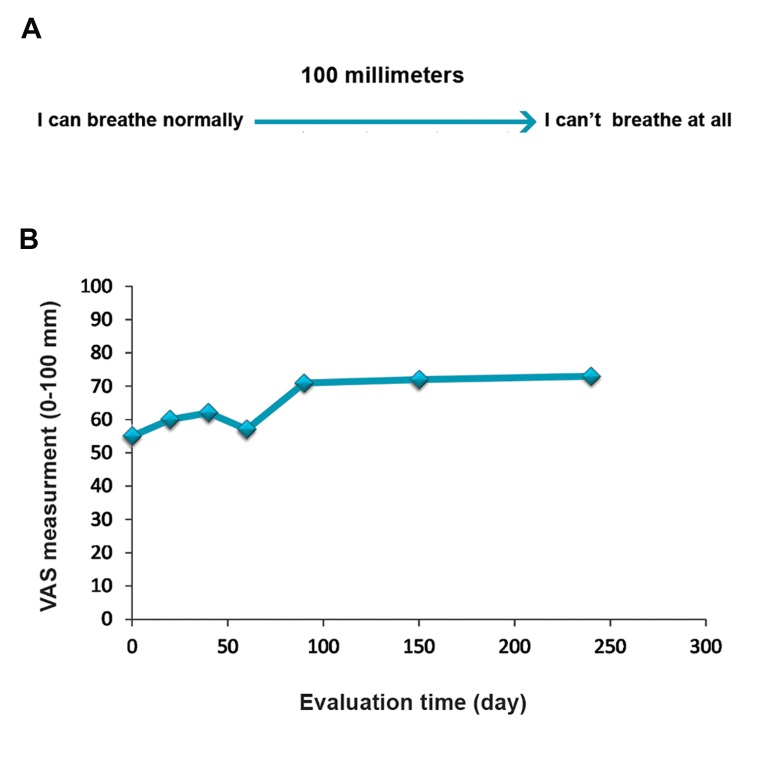
Evaluation of dyspnea visual analog scale before and after stem cell therapy A. Degree of breathing difficulty on the dyspnea Visual Analog Scale (VAS) by making a mark on a 100 mm uncalibrated horizontal line. The left end of the line is tagged as "I can breathe as I normally do" is scored as 0 and the right end of the line is scored as 100 and labeled as "I can’t breathe at all" and B. Evaluation of VAS before and after cell therapy.

Prior to initiation and after cell therapy, the patient was asked to indicate his current degree of breathing difficulty on the dyspnea Visual Analog Scale (VAS) by making a mark on a 100 mm uncalibrated horizontal line. The left end of the line is tagged as "I can breathe as I normally do" is scored as 0. The right end of the line is scored as 100 and labeled as "I can’t breathe at all" (Fig.8A). Accordingly, the VAS score is calculated by measuring in millimeters from the left end of the line to the point that the patient marks. Our result has shown that dyspnea improved gradually after stem cell therapy (Fig.8B).

**Fig.9:**
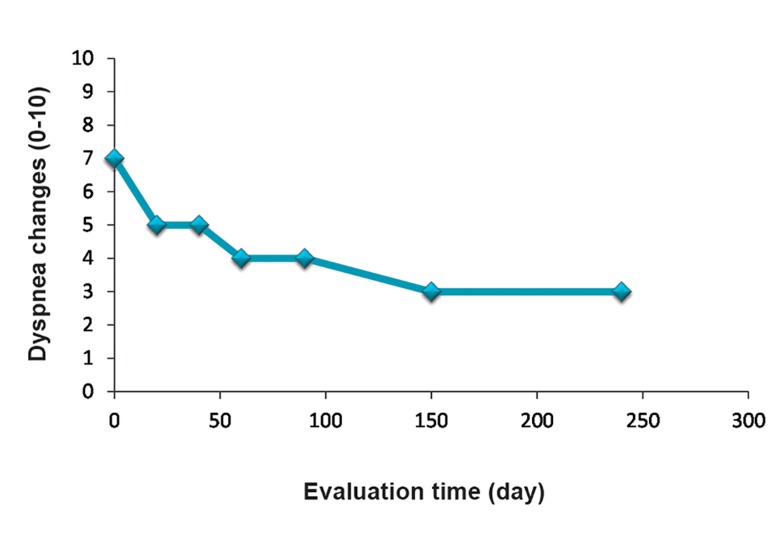
Evaluation of Borg Scale Dyspnea Assessment (BSDA) before and after cell therapy. BSDA showed improved respiratory parameters after each injection, especially at the 9 month follow up.

We observed a statistically significant difference after treatment in the BSDA scores from day 0 to month 9 (Fig.9).

**Table 1:** Results of other respiratory parameters after mesenchymal stem cell (MSC) injections


Evaluation time (day)	TLC%	RV%	RV/TLC%	Tlco (Hb)%	MEF25-75%

0	111	179	154	119	33
20	92	160	162	99	29
40	96	168	167	106	25
60	105	185	168	115	20
90	115	190	161	85	21
150	124	209	157	69	23
240	114	187	156	126	23


TLC; Total lung capacity, RV; Residual volume, Tlco; Transfer fac- tor, MEF; Maximum expiratory flow, and Hb; Hemoglobin.

## Discussion

Pulmonary injury induced by SM is very destructive because its duration is progressive and associated with a range of acute and chronic respiratory symptoms such as chronic bronchitis, asthma, and inflammatory airway (27). Drug therapy is not an effective treatment for these patients; thus, it is critical to find a better strategy for lung tissue regeneration (8). The therapeutic effects of stem cells in animal models and humans with different diseases have been shown (28). The beneficial effects of cell therapy include innocuous properties as well as low-cost and immunity compared to complications from transplants and graft rejection (29).

Application of autologous MSCs is a growing, potentially therapeutic method for a wide range of diseases. Clinical trials have demonstrated the safety of systemic MSC infusion. Thus far, no significant adverse effects have been noted in follow-up periods that lasted for several years in a variety of patient populations (30). MSCs from any of these sources have a range of anti-inflammatory effects that include release of anti-inflammatory molecules and activation of anti-inflammatory cellular pathways in different inflammatory environments (31). Most clinical investigations have used MSCs of bone marrow origin, however MSCs isolated from adipose tissue, placenta, and other sources are also being evaluated. The mechanisms of action by MSCs are not completely understood. In a lung damage model, MSCs have inhibited inflammation, decreased destructive changes, improved lung function, and reduced edema and fibrosis (32).

SM-exposed patients have a range of heterogeneous disorders that include both destructive airways and thickened bronchiolar walls with variable luminal mucus occlusion, as well as chronic pulmonary and systemic inflammation. Preclinical studies demonstrated the efficacy of both systemic and direct airway injections of MSCs. MSCs administered to mouse models with inflammatory and SM lung injuries reduced inflammatory cells and cytokines, as well as improved lung function and regeneration (33).

This study, for the first time, considered the therapeutic effects of MSCs on a patient with severe respiratory problems attributed to SM. Although we did not observe improvements in pulmonary function tests, a significant improvement existed in CAT, SGRQ, 6MWT, VAS, and BSDA scores. There were no observed adverse effects during the treatments and the patient felt satisfactory after each injection. Therefore, MSCs could be used 

as new tool for treatment of lung injuries caused by SM. However, a larger number of patients would be required to obtain significant results. We did not observe any clinical symptoms of pulmonary emboli during the MSCs infusions. These important observations demonstrated the safety of multiple MSC infusions in SM patients. Although we observed no significant effects from the MSC infusions on pulmonary function, the quality of life (QOL) indicators improved in our patient. According to the remodeling changes mentioned in previous studies, we have speculated that peribronchial fibrosis in these patients would not be quickly repaired and modified at this time. Thus, long-term follow up would be necessary. Large-scale trials with extended evaluation periods are needed to fully examine the potential effects of MSCs and other clinical assessments in these patients.

## Conclusion

Systemic administration of multiple doses of MSCs appears to be safe and improve 6MWT, CAT, SGRQ, VAS, and BSDA scores in SM-exposed patients with lung injuries. In addition, our patient has expressed satisfactory improvement in his legs and physical activities. These results provide an important, significant basis for further clinical investigations of MSCs in SM-exposed patients with lung injuries and other lung diseases. Further studies with larger number of SM-patients should be studied for MSCs therapy. 
